# Structural flexibility of the nicotinamide group of NADH in butanol dehydrogenase YqdH from *Fusobacterium nucleatum*

**DOI:** 10.1371/journal.pone.0338369

**Published:** 2025-12-17

**Authors:** Xue Bai, Ki Hyun Nam, Yongbin Xu

**Affiliations:** 1 Department of Bioengineering, College of Life Science, Dalian Minzu University, Dalian, Liaoning, China; 2 Key Laboratory of Biotechnology and Bioresources Utilization of Ministry of Education, College of Life Science, Dalian Minzu University, Dalian, Liaoning, China; 3 CAS Key Laboratory of Separation Science for Analytical Chemistry, Dalian Institute of Chemical Physics, Chinese Academy of Sciences, Dalian, Liaoning, China; 4 College of General Education, Kookmin University, Seoul, Republic of Korea; Federal University Dutse, NIGERIA

## Abstract

Butanol dehydrogenases (BDHs) are NAD(P)H-dependent oxidoreductases that catalyze the reversible conversion of butanol to butyraldehyde. These enzymes play essential roles in microbial butanol fermentation and show significant potential for biofuel synthesis and bioremediation. The crystal structures of BDHs from *Fusobacterium nucleatum* and *Thermotoga maritima* have clarified cofactor recognition and proposed reaction mechanisms. However, their distinct cofactor-binding modes and conformational differences in the substrate-binding cleft remain poorly characterized. In this study, we report the crystal structure of *Fusobacterium nucleatum* butanol dehydrogenase YqdH (FnYqdH) in a partially NADH-bound state. Electron density map analysis showed stable binding of the adenosine and diphosphate groups of NADH to the nucleotide-binding domain of FnYqdH. Conversely, the nicotinamide group was not observed, indicating that it was in an unbound state. Structural comparisons of FnYqdH complexed with either partial ADP or NADH revealed that the adenosine group is stabilized by hydrogen bonds with Thr143, Thr187, and Val184. Nicotinamide group binding induces positional and conformational changes in the diphosphate group of NADH. A comparative analysis of FnYqdH and TmBDH proteins revealed distinct conformational differences between their nucleotide-binding and catalytic domains, including variations in their substrate-binding metal ion sites. In particular, amino acid sequence and structural analyses of the BDH family revealed significant variability in the residues responsible for metal ion binding. Based on the observed flexibility of the nicotinamide group of NADH and the open conformation of FnYqdH, a potential reaction mechanism of FnYqdH is proposed. These findings offer valuable insights into the cofactor and substrate recognition within the BDH protein family.

## 1. Introduction

Butanol dehydrogenases (BDHs) are oxidoreductase enzymes that catalyze the reversible conversion of butanol to butyraldehyde, using NAD(P)H as a cofactor [1,2]. These enzymes play an essential role in microbial butanol biosynthesis, particularly within the acetone–butanol–ethanol (ABE) fermentation pathway utilized by *Clostridium* species [3–5]. Initially industrialized in the early 20th century, the ABE fermentation pathway remains a key metabolic route for bio-based butanol synthesis [6,7]. However, economic challenges have spurred continued efforts to optimize microbial strains and engineer the pathway for improved efficiency.

Butanol, a four-carbon alcohol derived from microbial fermentation, is a promising biofuel due to its high energy density, low hygroscopicity, and compatibility with the existing fuel infrastructure [8–10]. Beyond its use as a fuel, butanol and its derivatives are key intermediates in industrial chemistry, enabling the production of plastics, coatings, and pharmaceuticals [11]. This versatility underscores the significance of butanol dehydrogenases (BDHs), which not only facilitate butanol biosynthesis but also drive its further conversion into high-value chemicals [12,13]. In addition to biofuel production, BDHs are used in the enantioselective synthesis of chiral alcohols, fine chemical production, and bioremediation, reflecting their versatile catalytic potential [13]. Moreover, advances in protein engineering have led to the development of BDH variants with modified cofactor preferences and enhanced catalytic efficiency, enabling more efficient and sustainable biosynthetic pathways for butanol and other industrially important compounds [3,14].

BDHs exhibit considerable structural diversity within the same enzyme family, leading to variations in cofactor specificity, substrate specificity, and catalytic efficiency [15,16]. For instance, although the core structure of BDHs typically includes a nucleotide-binding domain with a conserved fold for NAD(P)(H) binding, significant conformational differences are observed within the substrate-binding cleft [17]. These structural variations are believed to play a key role in determining the catalytic outcomes of these enzymes, influencing their interactions with diverse aldehyde substrates and their efficiency in catalyzing oxidation/reduction reactions [17]. The structural relationship between cofactor binding and enzyme conformational states remains central to understanding the molecular mechanisms that regulate BDH activity [18]. Comprehensive structural analysis of BDH protein homologs is essential for elucidating the strategies underlying their metabolic flexibility and guiding their rational engineering for industrial biocatalysis.

While the biochemical properties of BDH enzymes are partially understood, the lack of high-resolution structural data has hindered a comprehensive understanding of their cofactor recognition and substrate-processing mechanisms. Therefore, determining the structures of representative BDH proteins in complex with their cofactors or substrates is essential for identifying the molecular factors that regulate their catalytic function.

The biochemical properties of various BDH proteins from several bacterial strains have been reported [19], but only a few high-resolution crystal structures have been determined—among which are those from *Fusobacterium nucleatum* (FnYqdH) and *Thermotoga maritima* (TmBDH) [20,21]. Both FnYqdH and TmBDH exhibit dehydrogenase activity with NAD(P)H and can catalyze not only butanal but also other aldehydes, such as propanal, pentanal, hexanal, heptanal, and octanal [20,21]. In both proteins, NAD(P)H binds to the nucleotide-binding domain; however, their cofactor-binding modes and the conformational relationships between the nucleotide-binding and catalytic domains differ significantly. In the FnYqdH–NADH complex, the substrate-binding cleft adopts an open conformation, positioning the nicotinamide group of NADH away from the metal-binding site responsible for substrate recognition [20]. Conversely, in the TmBDH–NADP complex, the substrate-binding cleft adopts a closed conformation, positioning the nicotinamide group of NADP closer to the metal-binding site [21]. These structural differences offer valuable insights into the conformational diversity among BDH proteins. Nevertheless, the functional relevance of the open conformation observed in NADH-bound FnYqdH—where the nicotinamide group remains distant from the catalytic site—remains unclarified.

To elucidate the molecular mechanism of FnYqdH, we conducted a structural analysis of both its native form and a partially NADH-bound complex. The binding of the adenosine and diphosphate groups of NADH and the nucleotide-binding domain were characterized, along with associated conformational changes. A comparative analysis of FnYqdH and TmBDH revealed key amino acid and structural differences in the cofactor-binding and active site regions. Based on these structural insights, we propose a putative reaction mechanism for FnYqdH. Collectively, these findings advance our understanding of molecular properties underlying BDH protein functions.

## 2. Materials and methods

### 2.1. Protein preparation

Protein expression and purification were performed as previously described [22]. In brief, the recombinant DNA containing the *yqdH* gene (UniProt: Q8R612), cloned into the pPRO-EX-HTA vector (Invitrogen, USA), was transformed into *Escherichia coli* BL21 (DE3). Cultures were grown in LB broth supplemented with 100 μg/mL ampicillin in a shaking incubator at 37°C until the optical density at 600 nm (OD_600_) reached 0.8–1.2. Protein expression was induced with 0.5 mM isopropyl β-D-thiogalactoside, followed by incubation at 30°C for 6–7 h. Cells were harvested by centrifugation and resuspended in lysis buffer (20 mM Tris-HCl, pH 8.0, 150 mM NaCl, and 2 mM β-mercaptoethanol). Cell lysis was achieved through sonication, and the lysate was clarified by centrifugation. The supernatant containing soluble protein was purified using Ni-NTA affinity chromatography. The target protein was eluted using a buffer containing 20 mM Tris-HCl (pH 8.0), 150 mM NaCl, 2 mM β-mercaptoethanol, and 250 mM imidazole. The hexahistidine tag was removed by incubating the protein with TEV protease overnight at 22°C. The sample was then further purified using ion exchange chromatography on a HiTrap Q column (GE Healthcare, USA). The eluted protein was concentrated and loaded onto a HiLoad Superdex 200 26/60 column (GE Healthcare, USA), which was equilibrated with a buffer containing 20 mM Tris-HCl (pH 8.0), 150 mM NaCl, and 2 mM β-mercaptoethanol. The purified protein was stored at −80°C until used for crystallization.

### 2.2. Crystallization

The protein solution was concentrated to approximately 20 mg/mL using a Vivaspin centrifugal concentrator (30 kDa cutoff, Millipore, USA). Crystallization was conducted using the hanging-drop vapor diffusion method at 6°C. A 1.5 μL sample of the FnYqdH solution was mixed with an equal volume (1.5 μL) of crystallization solution containing 0.1 M HEPES (pH 7.5) and 1.8 M ammonium phosphate. Crystals suitable for X-ray diffraction grew within 1 week. The dimensions of the FnYqdH crystals were approximately 100 × 100 × 20 μm^3^.

### 2.3. Data collection

X-ray diffraction data were collected at beamline BL17B of the Shanghai Synchrotron Radiation Facility (SSRF, Shanghai, China). The FnYqdH crystal was soaked for 1 min in the reservoir solution, which was supplemented with 25% (v/v) glycerol, 10 mM NADH, and 2 mM Co^2+^. The cryoprotected FnYqdH crystal was then mounted on a goniometer and cooled to 100 K using liquid nitrogen gas. Diffraction data were recorded with a Rayonix MX300 detector (Rayonix, LLC, Evanston, IL, USA). Diffraction images were indexed, integrated, and scaled using the HKL2000 program [23].

### 2.4. Structure determination

The phase problem was solved using the molecular replacement method with Phaser-MR, as implemented in PHENIX [24]. The crystal structure of native FnYqdH (PDB code: 6L1K) [20] served as the search model. Manual model building was conducted using COOT [25]. Structure refinement was performed using phenix.refine in PHENIX [24]. Water molecules were added during refinement using default parameters and manually validated against the electron density map. Cofactor binding was verified using a simulated annealing omit map. The geometry of the final model was validated using MolProbity [26]. Structural figures were generated using PyMOL [27]. The structure factors and coordinates were deposited in the protein data bank under accession codes 9UGT (FnYqdH^ADP^) and 9UGS (FnYqdH^NAT^). Data collection statistics are presented in Table 1.

**Table 1:** Data collection and refinement statistics.

Data collection	FnYqdH^NAT^	FnYqdH^ADP^
X-ray source	BL17B, SSRF	BL17B, SSRF
Space group	I222	I222
Cell dimension		
a, b, c (Å)	65.05, 78.91, 215.51	64.42, 79.35, 212.93
α, β, γ (°)	90.00, 90.00, 90.00	90.00, 90.00, 90.00
Resolution (Å)	50.0-2.50 (2.54.2.50)	50.0–2.70 (2.75–2.70)
Unique reflections	19608 (962)	14951 (725)
Completeness (%)	99.9 (99.9)	100.0(100.0)
Multiplicity	6.6(6.8)	12.4 (10.3)
I/sigma	11.0 (2.11)	11.875 (1.57)
CC1/2	0.963 (0.955)	0.947 (0.707)
**Refinement**		
Resolution (Å)	27.83-2.50	35.49-2.72
R_work_^a^	0.1849	0.2008
R_free_^b^	0.2373	0.2490
RMS deviations		
Bonds (Å)	0.008	0.009
Angles (°)	0.930	1.067
*B* factors (Å^2^)		
Protein	38.28	57.01
Water	36.83	44.61
ADP		80.53
Metal ion	86.51	103.96
Ramachandran plot		
Favored (%)	97.13	95.81
Allowed (%)	2.61	4.19
Disallowed (%)	0.26	0.00

^1^Values for the outer shell are given in parentheses. ^a^ R_work_ = Σ||Fobs| Σ |Fcalc||/Σ|Fobs|, where Fobs and Fcalc are the observed and calculated structure factor amplitudes, respectively. ^b^ R_free_ was calculated as R_work_ using a randomly selected subset (5%) of unique reflections not used for structural refinement.

### 2.5. Bioinformatics

Homologous proteins were identified using BLAST [28]. Multiple sequence alignment was conducted using Clustal Omega [29]. Structure-based sequence alignment was visualized using ESPript 3.0 [30]. The interaction distance between the cofactor and the protein was calculated using PLIP [31].

## 3. Results and discussion

### 3.1. Overall structure of the native and ADP-bound FnYqdH

**Fig 1:**
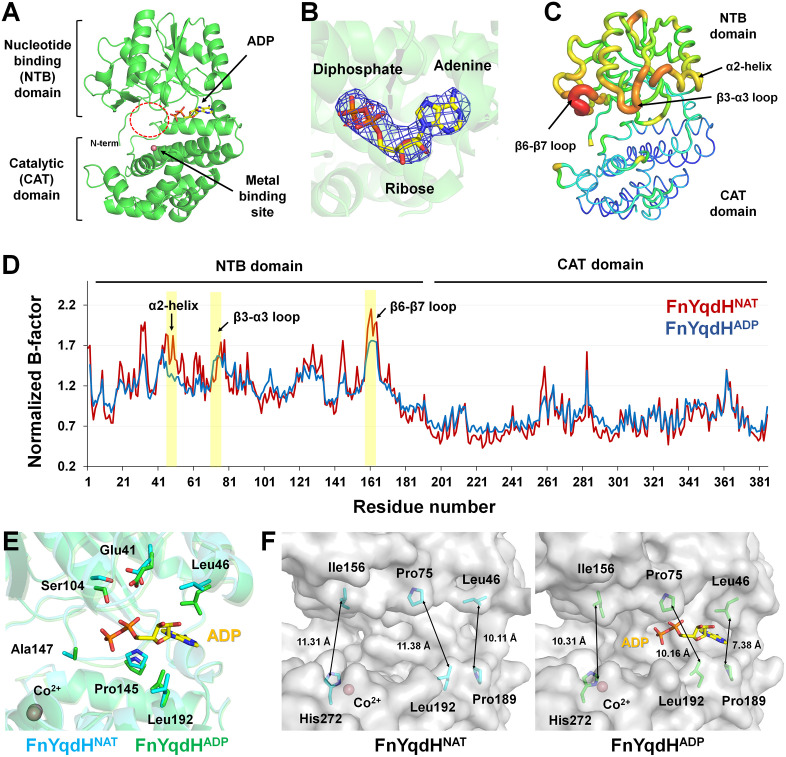
Crystal structures of FnYqdH^NAT^ and FnYqdH^ADP^. **(A)** Cartoon representation of FnYqdH^ADP^. **(B)** 2Fo-Fc (blue mesh, 1σ) and Fo-Fc (green mesh, 3 σ; red mesh, −3σ) maps for adenine and diphosphate from NADH in FnYqdH^ADP^. **(C)** B-factor putty representation of FnYqdH^ADP^. **(D)** Normalized B-factors of FnYqdH^NAT^ (red) and FnYqdH^ADP^ (blue). **(E)** Superimposition of the nucleotide-binding sites of FnYqdH^NAT^ and FnYqdH^ADP^. **(F)** Surface structure of the substrate- and nucleotide-binding clefts of FnYqdH^NAT^ and FnYqdH^ADP^.

In the previous study, we elucidated the overall structures of apo and NADH/Co^2+^-bound FnYqdH, and identified the amino acid residues involved in metal and cofactor binding through mutagenesis experiments. Although these findings offered valuable insights into the function of FnYqdH, the molecular mechanism by which substrate recognition and dehydrogenase activity occur in the open conformations of the cofactor-binding and catalytic domains has not yet been elucidated. Furthermore, the structural features of the cofactor- and metal-binding sites of FnYqdH have not yet been analyzed for conservation across other BDHs. To explore the molecular mechanism of FnYqdH, we determined the crystal structures of native and NADH-soaked FnYqdH at 2.50 Å and 2.70 Å resolution, respectively. The final models yielded R_work_/R_free_ values of 0.1849/0.2327 for native FnYqdH and 0.2008/0.2490 for NADH-soaked FnYqdH. The FnYqdH structure comprises an N-terminal nucleotide-binding domain (NTB: Met1-Tyr186) and a C-terminal catalytic domain (CAT: Asp190-Lys385) (Fig 1A). The NADH-soaked FnYqdH structure, molecular replacement was performed using the previously determined NADH-bound FnYqdH model, assuming a similar NADH-binding mode. The results revealed apparent electron density map for the adenosine and diphosphate binding sites; however, a significant negative Fo–Fc electron density was observed in the nicotinamide binding region. To avoid model bias, a simulated composite omit map of the NADH-binding site was analyzed. The omit map clearly revealed the presence of the adenine and diphosphate groups of the NADH molecule, but the nicotinamide group was not observed (Fig 1B). This finding indicates that the interaction between the ADP and diphosphate moieties of NADH and FnYqdH is stable, while the nicotinamide group of NADH interacts weakly with FnYqdH. Meanwhile, in the present study, we performed crystallization of FnYqdH with NADH and Co^2+^ under conditions that were nearly identical to those described in our previous report. However, unexpectedly, the newly obtained crystal structure revealed clear electron density only for the ADP portion of NADH, while the nicotinamide moiety was not visible, suggesting it was disordered. We believe that this discrepancy may be due to increased conformational heterogeneity of the nicotinamide group, potentially caused by subtle differences in the protein microenvironment, even under similar crystallization conditions. Similarly, in crystallographic studies, ligands or cofactors often adopt multiple conformations or orientations even under identical conditions. For example, in the crystal structure of glyceraldehyde 3-phosphate dehydrogenase (GAPDH), despite being derived from a single crystal, four D-G3H substrate molecules bound to each subunit exhibited different conformations, with one substrate showing a distinct binding position [32]. In the crystal structure of proteinase K complexed with triglycine, the triglycine molecule was found bound in two opposing orientations within the substrate-binding pocket [33]. These findings illustrate that even within a single crystal, ligands or cofactors can adopt heterogeneous conformational states in the active site or cofactor-binding site. Such structural variability may provide deeper insights into protein function, particularly in relation to substrate or cofactor recognition and catalytic mechanisms.

B-factor analysis showed that the NTB domain of both FnYqdH structures was more flexible than the CAT domain. Specifically, the B-factor values for the NTB domains were 48.73 Å^2^ for the native FnYqdH and 69.71 Å^2^ for the NADH-soaked FnYqdH, whereas those for the CAT domains were 28.90 Å^2^ for the native FnYqdH and 45.66 Å^2^ for NADH-soaked FnYqdH (Fig 1C). Notably, the flexibility of the NTB domain was most pronounced in the α2-helix (Glu45-Lys50), the β3-α3 loop (Gly71-Arg78), and the β6-β7 (Glu160-Lys162), all of which are located on the surface of the cofactor- and substrate-binding cleft. The B-factor value of the ADP molecule was 80.53 Å^2^, higher than that of the flexible α2-helix (71.63 Å^2^) and the whole protein, but lower than the flexibility observed in the β3-α3 loop (86.03 Å^2^) and β6-β7 loop (99.86 Å^2^) regions of the NTB domain (Fig 1C). Normalized B-factor analysis further revealed that, compared to native FnYqdH, the flexibility of the ADP-binding region and other flexible regions, such as the α2-helix, β3–α3 loop, and β6–β7 loop, was reduced in the NADH-bound FnYqdH (Fig 1D).

The overall fold of native FnYqdH (designated FnYqdH^NAT^) and the partially adenosine- and diphosphate-bound FnYqdH (designated FnYqdH^ADP^) was highly similar, with an r.m.s. deviation of 0.347 Å. However, significant conformational differences were observed in the cofactor-binding site and substrate-binding cleft between FnYqdH^NAT^ and FnYqdH^ADP^. When the CAT domains of FnYqdH^NAT^ and FnYqdH^ADP^ were superimposed, a shift in the main chain of the NTB domain, particularly near the ADP-binding region, became apparent, indicating that ADP-binding induces conformational changes in FnYqdH (Fig 1E). Specifically, the main chain of Pro145, located in the loop between the β5- and β6-strands of FnYqdH^ADP^, shifted by 1.9 Å toward the ADP molecule compared to FnYqdH^NAT^. Furthermore, the side chain of Leu192 in the CAT domain, near the ADP molecule, rotated by approximately 150°. When the NTB domains of FnYqdH^NAT^ and FnYqdH^ADP^ were superimposed, the side chains of Glu41, Leu46, and Ser104 rotated by approximately 90°, 62°, and 131°, respectively. These findings indicate that ADP-binding further induced conformational changes in both the cofactor- and substrate-binding clefts of FnYqdH. The substrate-binding cleft in FnYqdH^ADP^ was slightly narrower than that in FnYqdH^NAT^ (Fig 1F). In the adenine-binding region, the distance between the side chains of Leu46 and Pro189 was 7.38 Å in FnYqdH^ADP^ compared to 10.11 Å in FnYqdH^NAT^. In the ribose- and diphosphate-binding regions, the distance between the side chains of Leu75 and Leu192 was 10.16 Å in FnYqdH^ADP^ compared to 11.38 Å in FnYqdH^NAT^. In the region potentially corresponding to the nicotinamide group, the distance between the side chains of Ile156 and His272 was 10.31 Å in FnYqdH^ADP^, compared to 11.31 Å in FnYqdH^NAT^ (Fig 1F).

### 3.2. Nucleotide recognition of FnYqdH

**Fig 2:**
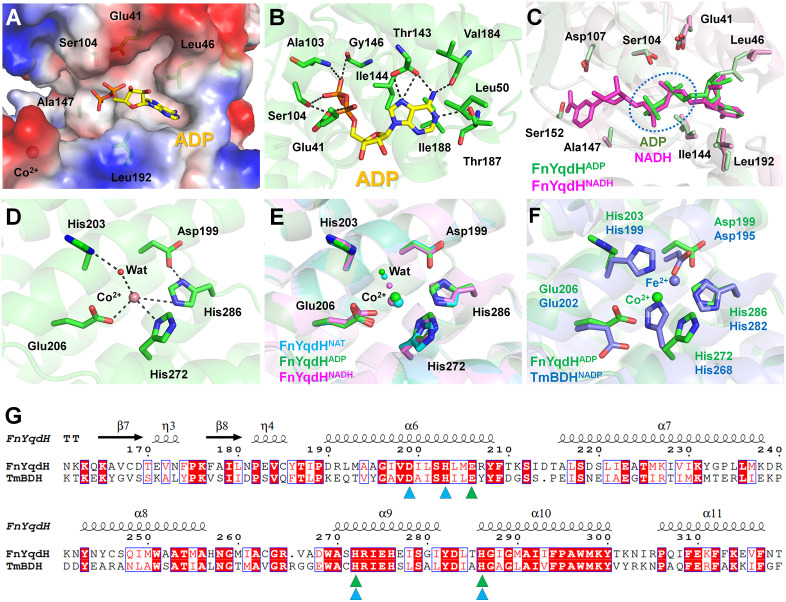
Structural analysis of nucleotide and metal ion-binding site in FnYqdH^ADP^. **(A)** Electrostatistic surface representation of the nucleotide-binding region of FnYqdH^ADP^. **(B)** Interactions between ADP diphosphate group and FnYqdH. **(C)** Structural superimposition of FnYqdH^ADP^ and NADH-bound FnYqdH (PDB code: 8I29). Metal ion-binding site of **(D)** FnYqdH^ADP^ and **(E)** TmBDH (PDB code: 1VLJ). **(F)** Superimposition of the metal-binding sites of FnYqdH and TmBDH. **(G)** Partial sequence alignment of metal-binding residues in FnYqdH (UniProt: Q8R612) and TmBDH (UniProt: Q9WZS7). Metal-binding residues in FnYqdH and TmBDH are indicated by green and cyan triangles, respectively.

In FnYqdH^ADP^, the adenine ring and diphosphate group of NADH are positioned on a predominantly hydrophobic, partially charged surface within the NTB domain (Fig 2A). The N1 atom of the adenine ring interacted with the OG1 atom of Thr187 at a distance of 3.39 Å (Fig 2B). The N6 atom of the adenine ring formed hydrogen bonds with the backbone carbonyl oxygens of Thr143 and Val184 at distances of 2.83 and 2.86 Å, respectively (Fig 2B). The N7 atom of the adenine ring formed additional hydrogen bonds with both the OG1 atom and the carbonyl oxygen of Thr143 at distances of 3.12 and 3.39 Å, respectively (Fig 2B). These structural analyses indicate that Thr143 and Thr187 are critical for cofactor recognition, consistent with our previous mutagenesis studies where the T143A and T187A mutants exhibited significantly reduced or abolished enzyme activity [20]. The adenosine ring is surrounded by hydrophobic residues (Leu46, Leu50, Ile144, Val184, and Ile188, all within 3.5–3.8 Å. The O1A atom of the α-phosphate group of NADH forms a hydrogen bond with the OE2 atom of Glu41 at a distance of 3.52 Å. The O2A atom of the α-phosphate group of NADH interacts with the OG atom of Ser104 at a distance of 2.80 Å (Fig 2B). The O3B atom of the β-phosphate group of NADH forms hydrogen bonds with the nitrogen atom of Ala103 and the carboxyl group of Gly146 at distances of 2.52 and 2.94 Å, respectively (Fig 2B). Conversely, the ribose molecule in ADP does not interact with FnYqdH and is exposed to the solvent region. The B-factor values for the adenine, ribose, α-phosphate, and β-phosphate of NADH were 67.30, 83.33, 90.98, and 94.95 Å^2^, respectively, indicating that the adenine group of NADH is relatively stable while the diphosphate group is more flexible.

To clarify the binding mode of partially bound NADH in FnYqdH (designated as FnYqdH^ADP^), the ADP-binding mode in FnYqdH^ADP^ was compared with that of fully bound NADH in the previously reported structure (PDB code: 8I29; designated as FnYqdH^NADH^). The structural superimposition of FnYqdH^ADP^ and FnYqdH^NADH^ showed an r.m.s.d. of 0.136 Å, indicating that the binding of the nicotinamide group of NADH to the NTB domain of FnYqdH does not significantly alter the overall conformation of the FnYqdH protein. However, notable differences were observed in the positioning of the ADP group of NADH and the conformations of the surrounding residues. The superimposition of FnYqdH^ADP^ and FnYqdH^NADH^ revealed that the adenine ring of NADH occupies an identical position in both structures; however, the positions of the ribose and phosphate groups differ (Fig 2C). The positional shifts of the C4, O2, and O3 atoms in the ribose molecule between FnYqdH^ADP^ and FnYqdH^NADH^ were 0.72, 0.76, and 1.37 Å, respectively. Upon superimposition of the FnYqdHADP and FnYqdHNADH structures, the phosphorus atoms of the α- and β-phosphate groups shifted by relative distances of 0.33 Å and 0.57 Å, respectively. The O1A atom of the α-phosphate and the O1B atom of the β-phosphate showed displacements of 0.82 and 0.98 Å, respectively, between the two structures. In FnYqdH^NADH^, the diphosphate group is positioned closer to the metal-binding site involved in the substrate binding than its position in FnYqdH^ADP^. This positional shift alters the interaction pattern with surrounding residues. In FnYqdH^ADP^, Ser104 interacts with oxygen atoms from α- and β-phosphate groups, whereas in FnYqdH^NADH^, it interacts only with the α-phosphate group (Fig 2C). These structural findings indicate that the ADP group of NADH is tightly bound to FnYqdH, whereas the nicotinamide group and phosphate regions exhibit weaker interactions. This further shows that NADH binding to FnYqdH is primarily stabilized by the adenine group. Furthermore, the observed differences in the position and conformation of the diphosphate groups between FnYqdH^ADP^ and FnYqdH^NADH^ imply that nicotinamide group binding to the NTB domain induces positional and conformational changes in the diphosphate group of NADH.

The metal ions in BDH play essential roles in enzyme catalysis through various mechanisms, including catalytic activity, substrate binding, and maintaining the structural stability of the enzyme [2,14,20]. The metal ion-binding site involved in substrate binding is located at the helical junction formed by the α8, α11−2, and α12 helices on the surface of the CAT domain of FnYqdH^ADP^. At this site, a Co^2+^ is coordinated by the OE1 atom of Glu206, the ND1 atom of His272, the NE2 atom of His286, and a water molecule, with coordination distances of 2.97, 2.91, 3.63, and 3.06 Å, respectively (Fig 2D). The water molecule interacting with Co^2+^ also interacts with the ND1 atom of His203 at a distance of 2.90 Å. Previous mutagenesis studies showed that substituting His203, Glu206, His272, and His286 with alanine completely abolished enzymatic activity [20]. The metal ion-binding configuration observed in this study is similar to that of both FnYqdH^NAT^ and the previously reported FnYqdH^NADH^ structure, indicating that metal coordination is unaffected by nucleotide binding or conformational changes between the NTB and CAT domains. Meanwhile, when compared with the apo state of FnYqdH (designated as FnYqdH^Apo^), in which the metal ion is absent, conformational changes in the metal-binding residues were observed. Superimposing the metal-binding sites of FnYqdH^NAT^ and FnYqdH^Apo^ shows that the side chain of His272 shifted toward the solvent region by 0.58 Å upon Co^2+^ binding.

Meanwhile, the metal ion-binding mode of FnYqdH differed significantly from that of the TmBDH protein (PDB code: 1VLJ). In TmBDH, Fe^3+^ is coordinated by the OD1 atom of Asp195 and the NE2 atoms of His199, His268, and His282 at distances of 2.09, 2.20, 2.28, and 2.30 Å, respectively—distinct from the Co^2+^ coordination observed in FnYqdH (Fig 2E). Despite this difference, structural superimposition of the CAT domains of FnYqdH and TmBDH revealed that the positions and conformations of the metal-binding His286 in FnYqdH and His282 in TmBDH were similar (Fig 2F). The Cα atom of the metal-binding His272 in FnYqdH aligns with that of His268 in TmBDH; however, their side chain conformations differ (Fig 2F). Similarly, the Cα position of the metal-binding Glu206 in FnYqdH corresponds to Glu202 in TmBDH; however, the side chain of Glu202 in TmBDH is oriented away from the metal-binding site and lies 7.15 Å from the Fe^3+^ site. In FnYqdH structures, the His202 residue interacts indirectly with the Co^2+^ site through a coordinating water molecule, whereas its structural equivalent, His199 residue in TmBDH, directly interacts with the Fe^3+^ site. Similarly, the Asp199 residue in FnYqdH is positioned approximately 6.0 Å from the Co^2+^ site, while its corresponding Asp195 residue in TmBDH binds with the Fe^3+^ site. Overall, although the amino acid sequences involved in metal ion-binding in FnYqdH and TmBDH are conserved (Fig 2G), their coordination modes differ significantly.

Meanwhile, our previous study found that the type of metal ion present significantly influences the enzymatic activity of FnYqdH [20]. Notably, Co^2+^ exhibited high catalytic efficiency, whereas Fe^3+^ showed relatively low catalytic efficiency, with less than 30% of the activity observed with Co^2+^ [20]. These results suggest that the nature of the metal ion, likely due to its electronic properties, plays a critical role in substrate recognition, conformational dynamics, and the geometry of the active site, thereby impacting the catalytic mechanism. This implies that structural changes in the active site may occur depending on the type of metal ion bound. Therefore, to fully understand the activity differences among BDHs associated with different metal ions, further structural studies of BDHs complexed with various metal ions, along with correlation analyses of their enzymatic activities, are necessary.

### 3.3. Structural comparison of the active sites of FnYqdH and TmBDH

**Fig 3:**
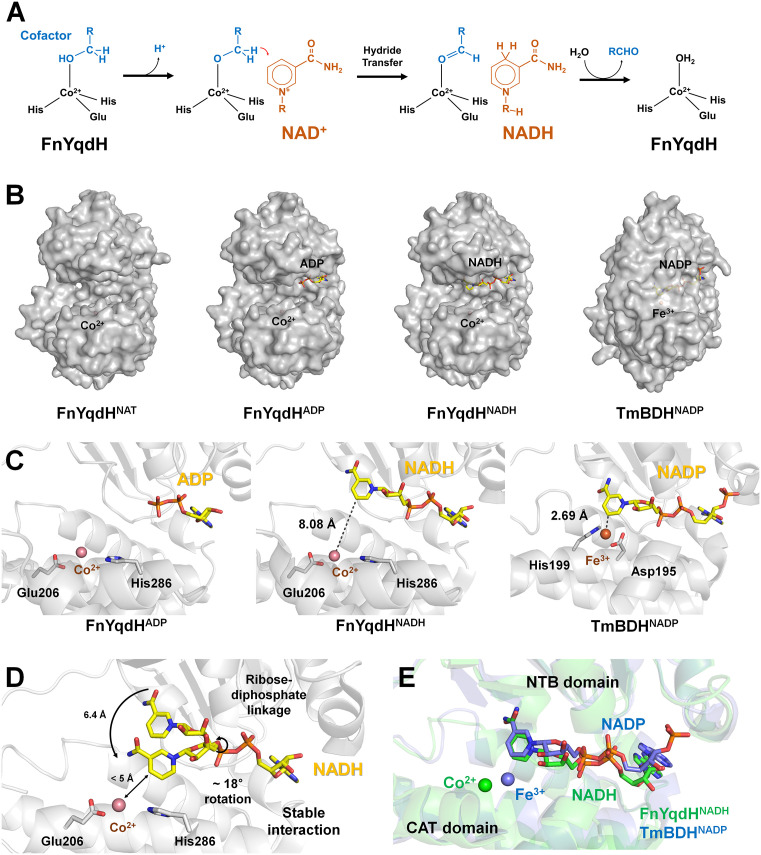
Conformation analysis and proposed conformational changes of FnYqdH during catalysis. **(A)** Proposed catalytic reaction mechanism of FnYqdH and other ADHs. **(B)** Conformational comparison of the NTB and CAT domains in FnYqdH^NAT^, FnYqdH^ADP^, FnYqdH^NADH^, and TmBDH^NADP^. **(C)** Close-up views of the active site and cofactor binding regions in FnYqdH^ADP^, FnYqdH^NADH^, and TmBDH^NADP^. **(D)** Hypothetical conformational change of NADH in the open form of FnYqdH. **(E)** Superimposition of the NB and CAT domains of FnYqdH^NADH^ onto the corresponding domains of TmBDH.

The proposed reaction mechanism of FnYqdH, inferred from previous studies and biochemical results [20], share key features with the typical reaction mechanism of the ADH family [34]. In the NADH-bound state of FnYqdH, substrate binding—such as butanol—to the metal ion within the CAT domain facilitates hydride transfer from the alcohol carbon atom to the oxidized nicotinamide group of NAD(P)^+^, yielding a Co^2+^-bound carbonyl product and NAD(P)H (Fig 3A). In the crystal structures of TmBDH and other ADHs, the nicotinamide group of NAD(P)H is positioned close to the metal-binding site involved in substrate binding, exhibiting a closed conformation between the NTB and CAT domains (Fig 3B). In TmBDH, the Fe^3+^ involved in the substrate-binding site is close to the C5N atom of the nicotinamide group of NADP at a distance of 2.63 Å (Fig 3C). Conversely, the FnYqdH^ADP^ and FnYqdH^NADH^ structures exhibited an open conformation between the NTB and CAT domains (Fig 3B). In FnYqdH^NADH^, the metal-binding site involved in substrate binding was positioned 8.08 Å away from the C5N atom of the nicotinamide group of NADH (Fig 3C). Meanwhile, in FnYqdH^ADP^, the nicotinamide group is not defined due to disordered electron density map, preventing the accurate measurement of the distance between this nicotinamide group and the active site. Therefore, FnYqdH does not appear to adopt a catalytically active structural state in which NADH and the metal ion are positioned in close proximity, as observed in other active ADHs, such as TmBDH complexed with NADP. Although the two crystal structures of FnYqdH^NADH^ and FnYqdH^ADP^ do not represent this active state—where the metal ion and the nicotinamide group of NAD(P)H are closely positioned—a functional dehydrogenase reaction requires these components to be within 3–5 Å to facilitate direct hydride and electron transfer from the substrate to NAD(P)^+^.

Given the observed flexibility of the nicotinamide group of NADH in FnYqdH, along with previous structural studies of TmBDH and other ADHs, we propose two potential reaction mechanisms that may position NADH and the metal ion within close proximity for the dehydrogenase reaction. First, FnYqdH may catalyze the dehydration reaction, even in its open conformation (Fig 3D). In this study, the nicotinamide amide group in FnYqdH was disordered, indicating that it may adopt multiple conformations. The β-N-glycosidic bond between nicotinamide and ribose, as well as the ribose–phosphate linkage in NADH, are single σ-bonds, which allow rotational flexibility of the nicotinamide group. Among the two rotatable σ-bonds in NADH, the ribose–phosphate linkage, involved in the nicotinamide group, may exhibit greater rotational flexibility. This is supported by the crystal structure of FnYqdH^ADP^, in which the nicotinamide–ribose portion was disordered. Building on these theoretical and experimental findings, we constructed a model to represent the rotation of the nicotinamide group in NADH bound to FnYqdH. When the σ-bond of the ribose–phosphate linkage associated with the nicotinamide group rotates approximately 18° toward the metal-binding site, the distance between the C4N atom of nicotinamide and the Co^2+^ ion reduces to less than 5 Å (Fig 3D). This proximity could potentially enable catalysis, even in the open conformation. Meanwhile, the crystal structure of FnYqdH^ADP^ provides valuable information about the flexibility of the nicotinamide group of NADH, it does not provide experimental evidence for the close proximity between the nicotinamide group of NADH and the metal binding site of FnYqdH. Consequently, the mechanistic interpretations proposed here should be considered hypothetical and require further investigation. To verify these proposed mechanisms and fully understand the molecular mechanism of FnYqdH, it is essential to determine the crystal structure of the enzyme in both cofactor- and substrate-bound states.

Second, FnYqdH may adopt a closed conformation between the NTB and CAT domains during the enzyme reaction, similar to TmBDH (Fig 3E). If FnYqdH can form a closed conformation between the NTB and CAT domains, it will bring NADH and the metal ion into closer proximity. When the NTB and CAT domains of FnYqdH^NADH^ were superimposed onto those of TmBDH, the NADH and metal ion-binding sites were positioned within 5 Å of each other (Fig 3E). Therefore, if FnYqdH can achieve this close conformation, the open conformation observed in the crystal structures of FnYqdH^ADP^ and FnYqdH^NADH^ likely represents an inactive state. These findings indicate that the structures of FnYqdH^ADP^ and FnYqdH^NADH^ do not support NADH as the key factor inducing the closed conformation between the NTB and CAT domains in FnYqdH. Instead, the observed open conformation in FnYqdH^NADH^ may result from crystallization conditions or crystal packing effects. To confirm this possibility, further crystallization experiments under different conditions are needed to capture the enzyme in an alternative conformation. In this study, the crystal structure of FnYqdH exhibited an open conformation between the CAT and NTB domains, similar to what was observed previously. This open state may tentatively explain the functional features of FnYqdH; however, it may not fully capture the structural basis of its catalytic mechanism. Therefore, molecular dynamics (MD) simulations may be required in future studies to gain deeper insights into the conformational dynamics of FnYqdH during catalysis. Such MD simulations could be conducted using both the experimentally observed open conformation and a modeled closed conformation of FnYqdH. These analyses may reveal conformational changes between the two domains, as well as provide information on cofactor binding modes and conformational changes that were not captured in the current structure.

## 4. Conclusion

Here, we report the crystal structure of butanol dehydrogenase FnYqdH in a partially NADH-bound state. This structure provides new insights into the structural flexibility of the nicotinamide group of NADH in FnYqdH, revealing its potential role in the enzyme’s catalytic mechanism. A detailed comparison with TmBDH highlights distinct structural features that contribute to the diversity within the BDH family. These structural analyses expand our knowledge of the properties of FnYqdH and further illustrate the structural diversity within the BDH family.
